# Interleukin 6/gp130 axis promotes neural invasion in pancreatic cancer

**DOI:** 10.1002/cam4.4823

**Published:** 2022-05-16

**Authors:** Hidetaka Suzuki, Shuichi Mitsunaga, Masafumi Ikeda, Takao Aoyama, Kazumi Yoshizawa, Masayuki Yamaguchi, Masami Suzuki, Minoru Narita, Toshikatsu Kawasaki, Atsushi Ochiai

**Affiliations:** ^1^ Division of Biomarker Discovery Exploratory Oncology Research & Clinical Trial Center, National Cancer Center Kashiwa Japan; ^2^ Laboratory of Pharmacotherapeutics Faculty of Pharmaceutical Science, Tokyo University of Science Tokyo Japan; ^3^ Department of Pharmacy National Cancer Center Hospital East Kashiwa Japan; ^4^ Department of Hepatobiliary and Pancreatic Oncology National Cancer Center Hospital East Kashiwa Japan; ^5^ Laboratory of Pharmacology and Therapeutics Faculty of Pharmaceutical Science, Tokyo University of Science Tokyo Japan; ^6^ Division of Functional Imaging Exploratory Oncology Research & Clinical Trial Center, National Cancer Center Kashiwa Japan; ^7^ Division of Cancer Genome Informatics Medicine Graduate School of Medicine, Osaka University Osaka Japan; ^8^ School of Pharmacy and Pharmaceutical Sciences Hoshi University Tokyo Japan

**Keywords:** mouse models, pancreatic cancer, pancreatic ductal adenocarcinoma, translational research

## Abstract

**Background:**

Nerve invasion (N‐inv) is an important prognostic factor in pancreatic ductal adenocarcinoma (PDAC). Elucidation of circulating N‐inv stimulators could provide deeper insights and novel perspectives for PDAC therapy. The interleukin (IL)‐6/gp130 axis was evaluated in this study as a candidate N‐inv stimulator.

**Methods:**

A human pancreatic cancer (PC) cell, Capan‐1, was confirmed to have the stimulant activity of IL‐6/gp130 axis through the evaluation of mRNA, cell surface protein and intracellular protein levels and chemotaxis and wound healing assay. The upregulation of IL‐6/gp130 axis was evaluated using tumor‐derived IL‐6 level and intratumoral pSTAT3 expression in N‐inv of murine sciatic nerves by intraneural injection of Capan‐1 cell (N‐inv model) and using resected pancreatic cancer tissue and clinical data from 46 PDAC patients.

**Results:**

mRNA and protein expressions of IL‐6 and IL‐6 receptor were found in whole cell lysate and condition medium from PC cell. Cell surface protein expression of gp130 were clearly detected on PC cell. IL‐6 promoted migration and chemotaxis of PC cell. Serum IL‐6 and tumoral IL‐6 mRNA levels in N‐inv model mice were significantly higher than those in subcutaneous tumor mice (*p* = 0.004 and *p* = 0.002, respectively). Silencing of IL‐6 and gp130 on PC cell and administration of an anti‐IL‐6 receptor antibody, tocilizumab, suppressed N‐inv, compared to each control (*p* = 0.070, *p* = 0.118 and *p* = 0.122, respectively). In PDAC patients, the high‐N‐inv group showed poor prognosis (*p* =0.059) and elevated serum levels of IL‐6 and C‐reactive protein, synthesis of which is promoted by IL‐6, compared to those in the low‐N‐inv group (*p* = 0.006 and *p* = 0.075, respectively). Tumoral gp130 expression at N‐inv was higher than that in the primary pancreatic tumor (*p* = 0.026).

**Conclusion:**

Biological activity of IL‐6/gp130 axis promoted N‐inv in murine model and was upregulated in PDAC patients with severe N‐inv. This study is the first evidence that the IL‐6/gp130 axis offers a potential therapeutic target in PDAC with N‐inv.

## Graphical abstract

Nerve invasion (N‐inv) is an important prognostic factor in pancreatic ductal adenocarcinoma (PDAC). To elucidate circulating stimulators for nerve invasion, the authors evaluated the stimulant activity and the inhibitory effects of IL‐6/gp130 axis. Biological activity of IL‐6/gp130 axis promoted nerve invasion in murine model and was upregulated in PDAC patients with severe N‐inv. This study is the first evidence that the IL‐6/gp130 axis offers a potential therapeutic target in PDAC with N‐inv.

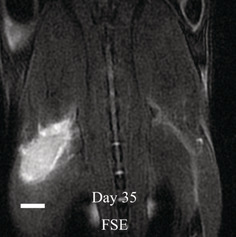

## 1 INTRODUCTION

Nerve invasion (N‐inv) is a common invasive behavior and has been confirmed as an important prognostic factor in pancreatic ductal adenocarcinoma (PDAC).^1^, ^2^, ^3^ N‐inv can cause systemic inflammation,^4^ severe pain,^5^, ^6^ cachexia,^7^ and worsened quality of life. A recent study revealed that progression of N‐inv was dependent on phosphorylated signal transducer and activator of transcription‐3 (pSTAT3) in an N‐inv model using the MiaPaCa‐2, human pancreatic cancer (PC) cell.^8^ MiaPaCa‐2 exhibited a dose–response relationship between interleukin (IL)‐6 exposure and STAT3 activation.^9^ The IL‐6 superfamily, which includes IL‐6, leukemia inhibitory factor (LIF) and oncostatin M (OSM), interacts gp130 and activates Janus kinases (JAKs), and STAT pathway.^10^ We have speculated that the IL‐6 superfamily/gp130 axis promotes N‐inv via STAT3.

N‐inv model in our previous study^11^ was made by inoculating human PC cells into the sciatic nerve of mice with severe combined immunodeficiency. Our N‐inv model mimic morphological aspects and symptoms of N‐inv in PDAC patients.^4^, ^6^ This N‐inv model showed high invasive capacity and the molecular signature of STAT3 activation,^8^, ^11^ and was considered an appropriate model for elucidating IL‐6 superfamily/gp130 axis in N‐inv. As the results of this study showed that the IL‐6/gp130 axis promoted N‐inv in murine model, tocilizumab, an anti‐human IL‐6 receptor (IL‐6R) antibody, was administered to N‐inv model. Tocilizumab binds both membrane‐bound IL‐6R and soluble IL‐6R (sIL‐6R) and suppresses the JAK/STAT pathway by inhibiting the dimerization of gp130 molecule.^12^, ^13^

This study was planned to evaluate: (a) stimulatory values of IL‐6, LIF, and OSM for migration of PC; (b) inhibitory effects of ligand/receptor axis on N‐inv; and (c) the relationship between N‐inv and expression levels of ligand/receptor axis in patients with PDAC.

## 2 MATERIAL AND METHODS

### 2.1 Cells

Two human PC cell lines, Capan‐1 (RRID: CVCL_0237) and BxPC‐3 (RRID: CVCL_0186) were obtained from American Type Culture Collection (Manassas, VA) and were incubated at 37°C under an atmosphere of 5% CO_2_ in air. These cells were propagated and subcultured according to the recommended ATCC protocol.

### 2.2 Mouse model

The N‐inv model using Capan‐1, subcutaneous tumor (SC) model using Capan‐1, and PBS model were produced as previously described.^11^ Briefly, 6‐week‐old SCID mice were used in our study. After induction of anesthesia with 4–5% isoflurane in oxygen (O_2_), mice were maintained in 2%–3% isoflurane anesthesia via a nose cone throughout the operation. After the left sciatic nerve of each mouse was exposed at the level of femur, 2.5 μl of phosphate buffered saline (PBS) containing 2.5 × 10^4^ Capan‐1 cells was injected into the sciatic nerve using a micro‐syringe (Hamilton) and 30‐gauge needle (Becton Dickinson and Company). AG490, an inhibitor of JAK2, at a dose of 0.5 mg/kg (Calbiochem; *n* = 6) or dimethyl sulfoxide (DMSO) vehicle (1%/1 ml/body, *n* = 4) was administered to N‐inv mice by intraperitoneal (i.p.) injection from day 14 to day 28, daily. Anti‐IL‐6R antibody (*n* = 6; Chugai Pharm. Co.), immunoglobulin (Ig)G (*n* = 4; Sigma‐Aldrich), anti‐mouse IL‐6R antibody (*n* = 4; Chugai Pharmaceutical Co.), or rat IgG (*n* = 5; Sigma‐Aldrich), each at a dose of 5 μg/g body weight, was administered to N‐inv mice by i.p. injection twice a week from day 7 to day 21. After euthanasia with continued exposure to 5% of isoflurane until respiration ceases and death ensues, mice were sacrificed 2 h after the last dose. The humane endpoints were defined as follows: (a) loss of ≥30% of the maximum weight; (b) profound abnormal behavior; (c) skin ulcer with tumor necrosis. If mice reach the humane endpoints, we will humanely euthanize with continued exposure to 5% of isoflurane. The critical organs (i.e., brain, heart, or lungs) were harvested immediately after euthanasia as the acceptable alternative method to confirm animal death in accordance with the American Veterinary Medical Association guidelines. All animal experiments were carried out in accordance with the Guidelines of the Animal Care and Use Committee of the National Cancer Center.

### 2.3 MRI

The three N‐inv mice were examined on days 21, 28, and 35 following inoculation of PC cells. All images were taken with a 3‐T whole‐body scanner (Signa HDx; GE Medical Systems). A body coil and solenoid coil developed specifically for research use (35 mm in diameter) were used for radiofrequency transmission and signal reception, respectively. Mice were anesthetized with 1%–2% isoflurane with a 1:1 gas mixture of O_2_ and nitrous oxide (N_2_O) administered via nose cone. After induction of anesthesia, the mouse was fixed to a cradle in the prone position and placed in the center of the solenoid coil. Coronal T_2_‐weighted images of the sciatic nerve were acquired using fat‐suppressed fast‐spin‐echo (FSE) sequence. T_2_‐weighted images (repetition time/effective echo time = 3500/60 ms, echo train length = 8) were obtained with a slice thickness of 1 mm and no inter‐slice gap, a 40‐mm field of view, and a 256 × 256 matrix. The number of acquisitions were set at 4.

### 2.4 Migration assay

Capan‐1 cells (3.0 × 10^5^ cells) were incubated on 24‐well plates for 24 h, with cells serum‐starved overnight. The resulting cell monolayer was scraped in a straight line to create a scratch using the tip of a 1000‐μl pipette. After creating a scratch, the cells were washed twice using PBS to remove nonadherent cells and serum‐free medium was added. Photographs were taken under a microscope (ECLIPSE TE300; Nikon) with a 10× objective lens at 0 and 24 h. Migration distance was analyzed using Image J software (National Institutes of Health). Each experiment comprised triplicate trials. Scratch healing distance was calculated as follows: scratch width at 0 h––scratch width at 24 h.

Alternatively, cell mobility was measured as the cell confluence rate of the wound area using the Incucyte® Scratch Wound Assay (Essen Bioscience), in accordance with the protocol described by the manufacturer. Briefly, Capan‐1 cells (6.0 × 10^4^ cells) were incubated with a culture medium for 24 h in 96‐well ImageLock cell migration plates (Essen Bioscience). The confluent cell monolayer was then wounded with a 96‐pin WoundMaker (Essen Bioscience) and exposed to serum‐free medium with/without recombinant IL‐6 (rIL‐6) and rIL‐6Rα (both from R&D Systems). Cell mobility was measured at 24 h after scratch creation by selecting the “scratch wound” scan type.

### 2.5 Chemotaxis assay

Chemotaxis assay was performed according to the protocol from the manufacturer (BD Biosciences). Briefly, Capan‐1 cells (2.0 × 10^5^ cells per insert) were seeded in Falcon® Permeable Support for 24‐well Plate with 8.0 μm Transparent PET Membrane (BD Biosciences) and migrated toward the basal chamber containing chemoattractant. Migrated cells were stained with Mayer's hematoxylin (Muto Pure Chemicals) and evaluated in nine randomized fields at 20× magnification. Each experiment consisted of triplicate trials.

### 2.6 Immunohistochemistry (IHC)

After microwave heating in 0.01‐M citrate buffer or high‐pH buffer, slides were incubated with listed antibodies (Table. S1) at 4 °C overnight. Expression of pSTAT3 was evaluated in the proximal invasive front or/and distal invasive front at 40× magnification and was quantified by labeling index (LI): {(number of pSTAT3‐positive cancer nuclei)/(number of all cancer nuclei) × 100}. The proximal and distal invasive fronts were defined as the tip of intraneural tumor positioned proximal and distal from injection point, respectively. The injected sciatic nerve of each mouse was harvested, fixed in 4% paraformaldehyde overnight at 4°C, and embedded in paraffin. Serial 3‐μm sections were cut along the nerve route. One of the longitudinal sections was stained with hematoxylin and eosin, and the proximal and distal invasive front were evaluated.

Invasive fronts of primary tumor and N‐inv in resected specimens of human PDAC of pancreas head were evaluated for IHC analysis of gp130 expression. The invasive front of the primary tumor was defined as peripheral to the whole primary tumor and in the most severe extent of tumor into the surrounding tissue. Three random parts per section at the invasive front of the primary tumor were evaluated at 20× magnification. The manner of selection for the invasive front of N‐inv was as previously described.^2^ The percentage of gp130‐positive area was calculated as the ratio of the positive area per measured area using the automeasure function of Axio Vision version 4.7.1 software (Carl Zeiss). The mean in each patient was then recorded.

### 2.7 ELISA

Levels of human IL‐6 and IL‐6R in conditioned media of cultured cells were quantitatively determined in duplicate using an ELISA kit in accordance with the protocol from the manufacturer (both R&D Systems). Culture supernatants were collected and filtered through a micro‐filter (pore size, 0.22 μm; Millipore).

### 2.8 Western blot

The method of western blotting was as previously described.^11^ Briefly, 20 μg of total cell protein were electrophoresed in a 4%–20% SDS‐polyacrylamide gel and immunoblotted onto a polyvinylidene difluoride membrane (Bio‐Rad Laboratories, Hercules, CA). Membranes were blocked using 5% nonfat dry milk in Tris‐buffered saline with Tween 20 (TBST) (Tris–HCl, pH 7.5, Tween 0.1%) or Blocking One‐P (Nacalai Tesque) for 1 h at room temperature and were incubated with the listed primary antibodies (Table. S1) at 4°C overnight. Immunoblots were developed using enhanced chemiluminescence (ECL) Prime Western Blotting Detection Reagent (Cytiva). The optical density (OD) of the bands was determined using ImageJ software (National Institutes of Health). Normalized expression of phosphorylated protein was calculated with the following equation: {(OD of phosphorylated protein)/(OD of total protein)}. The expressions of phosphorylated protein at every time point were calculated by regarding the OD at 0 min as 1.00. β‐actin was used as loading control for western blotting.

### 2.9 Fluorescence‐activated cell sorting (FACS)

Each trial used 200,000 cells. Peripheral blood mononuclear cells (PBMCs) were isolated from a whole blood sample from two researchers (H.S. and S.M.) using a Vacutainer® CPT™ Cell Preparation Tube with Sodium Heparin N (BD Biosciences). The use of blood sample from researchers was approved by the National Cancer Center Ethics Committees. PBMCs were used for measurement immediately after isolation. Capan‐1 was stained with mouse anti‐human gp130‐phycoerythrin (PE) (R&D Systems) and mouse anti‐human CD126‐fluorescein isothiocyanate (Diaclone). Capan‐1 stained with mouse anti‐human gp130‐PE was stained with anti‐mouse IgG1‐allophycocyanin. Flow cytometry was performed on a BD Accuri™ C6 Plus Flow Cytometer (BD Biosciences). Capan‐1 and PBMCs were gated by FSC/SSC scatter characteristics. Positivity threshold was determined as a 99% percentile cutoff based on isotype IgG in each cell. Data were analyzed with C6 Plus software version 1.023.1 (BD Biosciences).

### 2.10 Real‐time reverse transcription‐PCR (RT‐PCR)

RNA extraction and real‐time RT‐PCR were performed as described previously.^11^ Primer sequences were designed by Takara Bio and shown in Table S2. Relative gene expressions were determined using the 2–ΔΔCt method with *GAPDH* as the internal reference gene in each sample.^14^

### 2.11 siRNA

Capan‐1 cells (3 × 10^5^ cells) were plated on 6‐well plates for 48 h prior to siRNA treatment, as described previously.^11^ The siRNAs targeted IL‐6R (sense, ′CGACUCUGGAAA CUAUUCATT‐3′; antisense, 5′‐UGAAUAGUUUCCAG AGUCGTG‐3′; Thermo Fisher Scientific, Waltham, MA) and gp130 (sense, 5’‐GGCAUGCCUAAAAGUUACUT T‐3′; antisense, 5′‐UGAAUAGUUUCCAGAGUCGTG‐3′; Thermo Fisher Scientific). Silencer® Negative Control #1 siRNA (Thermo Fisher Scientific) was used as control siRNA.

### 2.12 Short hairpin RNA (shRNA)

Continuous RNA interference using lentivirus (LV)‐based shRNA was used as previously described.^11^ Vectors were constructed using standard cloning procedures. The targeting sequence and shRNA sequence are provided in Figure S1.

### 2.13 Radiological evaluation of N‐inv in PDAC patients

Radiological N‐inv was then evaluated in 46 patients (28 women, 18 men; median age, 65.5 years; range, 44–85 years; primary tumor sites in pancreatic head/body/tail, 21/20/5) with pathologically confirmed advanced PDAC who had been treated at National Cancer Center Hospital East, Japan between June 2008 and March 2011. Serum levels of IL‐6 and sIL‐6R were measured with ELISA. Criteria for radiological N‐inv on CT images and methods of analysis were as previously reported.^4^ Briefly, according to National Comprehensive Cancer Network Clinical Practice Guidelines in Oncology: Pancreatic Adenocarcinoma,^15^ the degree of radiological N‐inv was classified as high or low N‐inv according to the severity of perivascular soft tissue (PVST) around the superior mesenteric artery (SMA) and the celiac artery (CeA). Low radiological N‐inv was defined as PVST that encircled neither the SMA nor the CeA completely. The complete encirclement of PVST around the SMA or the CeA was defined as high radiological N‐inv.

### 2.14 Specimens of PDAC patients for IHC

The specimens obtained through pancreaticoduodenectomy were sectioned along the long axis of the plexus pancreaticus capitalis between September 1992 and January 2004 in our institution.

### 2.15 Data analysis and statistics

Results are reported as mean ± SD unless stated otherwise. A two‐tailed unpaired Student's *t*‐test or ANOVA and the post hoc test was used to evaluate differences in the various parameters. A value of *p* < 0.05 was considered significant. Statistical analysis was performed using JMP® version 11 (SAS Institute, Cary, NC).

## 3 RESULTS

### 3.1 Stimulant activity of IL‐6 superfamily on human PC cell migration in vitro

**FIGURE 1:**
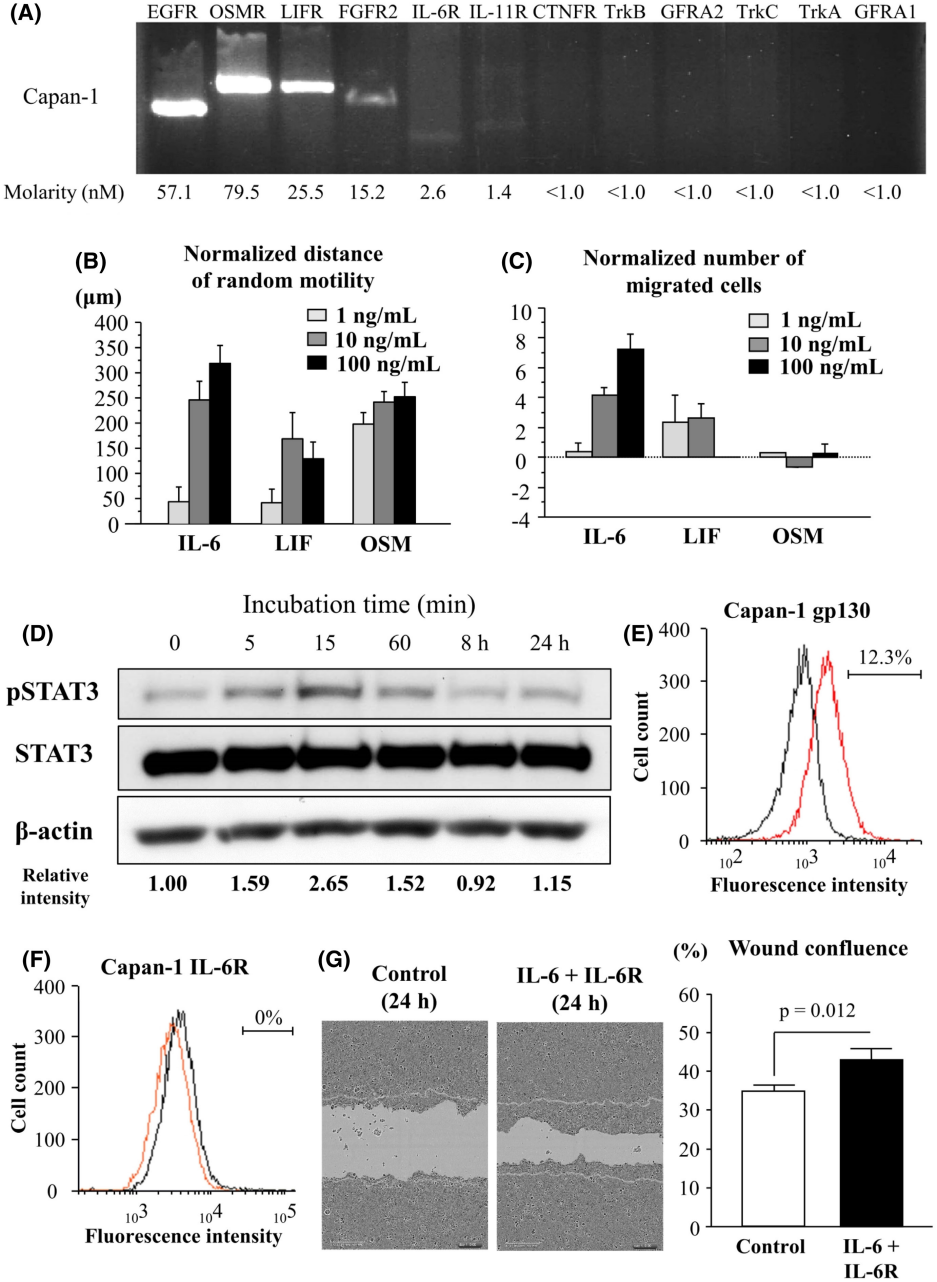
Stimulant activity of IL‐6, LIF, and OSM on pancreatic cancer cell migration. (A) Gene expressions of receptors related to neurotrophic factors are evaluated in human pancreatic cancer cell lines Capan‐1. Capan‐1 have a high ability to invade neural tissue. The mRNA expressions of target genes are expressed as molarity. (B, C) Influences of each ligand on migration and invasion are measured by means of wound healing assay (B) and chemotaxis assay (C), respectively. Imaging was conducted at 0 and 24 h under light microscopy (*n* = 9 each). Wound healing is quantified as the normalized distance of random motility. Results of chemotaxis assay are presented as normalized number of migrated cells 24 h after start. (D) Western blot analysis of pSTAT3 under IL‐6 stimulation in Capan‐1. Cells were serum‐starved for 24 h and pretreated with IL‐6 (10 ng/ml) stimulation or recombinant free medium for the indicated times. Cells were then harvested and run for western blot analysis as described in the Materials and Methods. Densitometry analyses of pSTAT3 normalized to tSTAT3 were shown in the bottom of bands. (E–F) Cell surface expressions of gp130 protein (in red) (E) and IL‐6R protein (in orange) (F) analyzed by flow cytometry from cultivated Capan‐1. (G) Comparison of wound confluence at 24 h after scratch of cultured face between IL‐6 + IL‐6R group (right picture) and control group (left picture). The image is shown at 10× magnification. The right graph shows wound confluence in each group

Capan‐1 cells (Figure 1A) and BxPC3 cells (Figure S6) clearly possessed mRNA of IL‐6R, OSMR, LIFR, and gp130. To investigate the stimulant activity of IL‐6, LIF or OSM, migration and chemotaxis assays using rIL‐6, rLIF and rOSM were performed in PC cell lines, Capan‐1 and BxPC3. IL‐6 was the strongest capability to promote migration (1 ng/ml vs. 10 ng/ml vs. 100 ng/ml [mean ± SD]: IL‐6 43.33 ± 89.08 mm vs. 246.39 ± 127.46 mm vs. 319.72 ± 117.87 mm, *F* (2, 30) = 15.509, *p* < 0.0001; LIF 42.22 ± 80.47 mm vs. 168.89 ± 158.27 mm vs. 128.89 ± 98.36 mm, *F* (2, 24) = 2.748, *p* = 0.084; OSM 197.78 ± 72.13 mm vs. 241.11 ± 62.41 mm vs. 251.11 ± 90.80 mm, *F* (2, 24) = 1.251, *p* = 0.304; Figure 1B) and chemotaxis (1 ng/ml vs. 10 ng/ml vs. 100 ng/ml [mean ± SD]: Capan‐1, IL‐6 0.40 ± 1.30 cells vs. 4.20 ± 1.14 cells vs. 7.20 ± 2.30 cells, *F* (2, 12) = 20.988, *p* = 0.0001, LIF 2,33 ± 3.21 cells vs. 2.67 ± 1.53 cells vs. 0.00 ± 1.00 cells, *F* (2, 6) = 1.390, *p* = 0.319, OSM 0.33 ± 0.00 cells vs. −0.67 ± 0.00 cells vs. 0.33 ± 1.00 cells, *F* (2, 6) = 3.000, *p* = 0.125; BxPC3, IL‐6 −1.00 ± 0.89 cells vs. 2.80 ± 4.09 cells vs. 9.40 ± 6.34 cells, *F* (2, 12) = 7.199, *p* = 0.009, LIF 0.33 ± 0.58 cells vs. −0.33 ± 0.58 cells vs. 0.00 ± 1.00 cells, *F* (2, 6) = 0.600, *p* = 0.579, OSM 0.33 ± 2.65 cells vs. 2.00 ± 0.58 cells vs. 3.67 ± 2.89 cells, *F* (2, 6) = 1.596, *p* = 0.278; Figure 1C, Figure S2) in Capan‐1 and BxPC3. The stimulatory effects of rIL‐6 were evidently strongest at 15 min after beginning IL‐6 stimulation in Capan‐1, respectively (0 min vs. 5 min vs. 15 min vs. 60 min vs. 8 h vs. 24 h: 1.00 vs. 1.59 vs. 2.65 vs. 1.52 vs. 0.92 vs. 1.15; Figure 1D). Human PBMCs are known to detect IL‐6R and gp130 on cell surfaces,^16^ anti‐IL‐6R and gp130 antibodies were apparently workable and effective from flow cytometry of PBMCs (Figure S3A,B). Cell surface expression of gp130 was found on Capan‐1, but not IL‐6R (Figure 1E,F). Since IL‐6R protein was faint on cell surfaces of PC cells, soluble IL‐6R was needed to transduce the intracellular signal of IL‐6 in Capan‐1. To confirm secretion of IL‐6 and sIL‐6R from Capan‐1 cells, the levels of IL‐6 and sIL‐6Ra in the conditioned media of the cultured cells were quantitatively determined by ELISA. The concentrations of IL‐6 and sIL‐6R were 80.27 ± 0.92 pg/ml and 3.72 ± 0.22 pg/ml, respectively. Moreover, co‐stimulation of IL‐6 and IL‐6R significantly promoted migration in Capan‐1 (IL‐6 + IL‐6R vs. control: 43.26 ± 12.88 vs. 35.02 ± 7.52, *p* = 0.012; Figure 1G).

### 3.2 Expression of IL‐6 and pSTAT3 in N‐inv model

**FIGURE 2:**
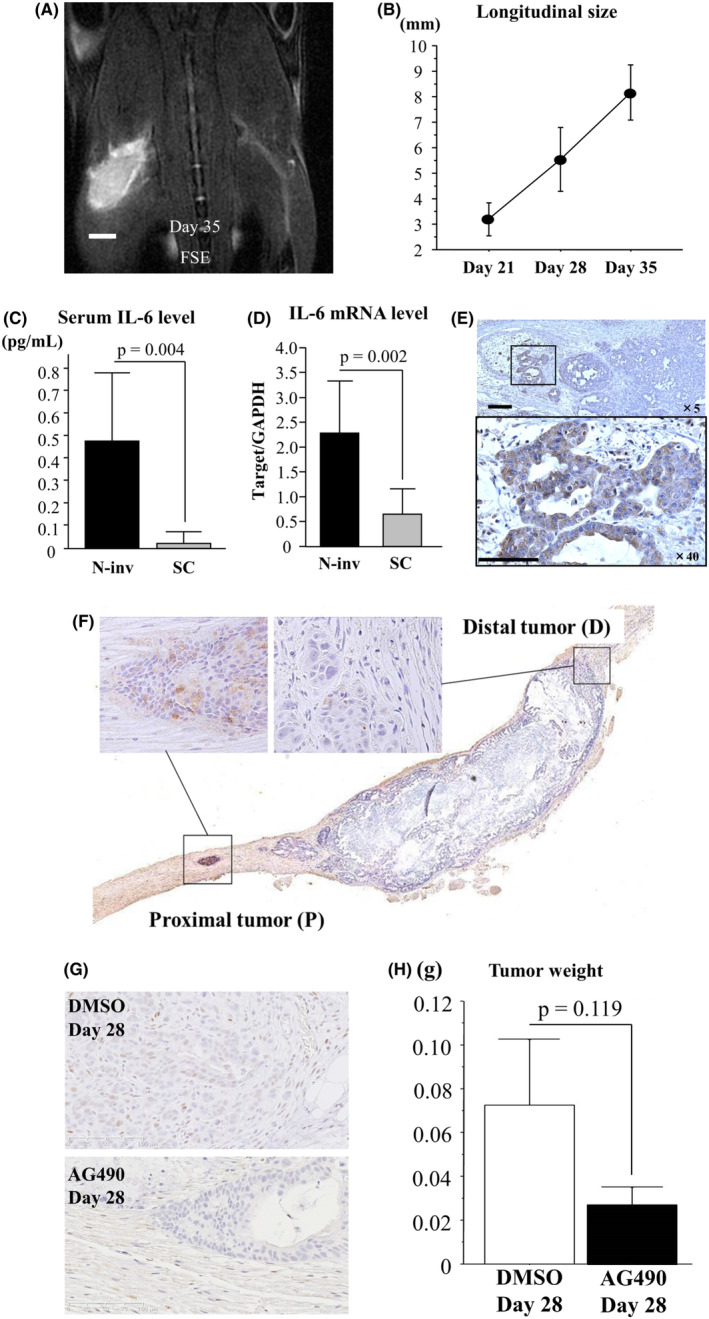
Growth of N‐inv tumor and expression of IL‐6 and pSTAT3 in N‐inv model. (A) Visualization increases in longitudinal tumor size on coronal FSE image of N‐inv model mice over time up to day 35. Scale bar: 2 mm. (B) Longitudinal size of N‐inv measured on days 21, 28, and 35 after Capan‐1 cell inoculation (*n* = 3). (C) Serum concentration of human IL‐6 in N‐inv (*n* = 8) and SC (*n* = 6) mice. Serum levels are quantified with ELISA on day 28 after Capan‐1 cell inoculation. (D) Expression of IL‐6 mRNA in N‐inv (*n* = 7) and SC (*n* = 6) tumors on day 28 after Capan‐1 cell inoculation. Expression of IL‐6 mRNA is normalized to that of GAPDH mRNA. (E) IHC image for IL‐6 of invasive tumor in the left sciatic nerve of N‐inv mice at day 28 after Capan‐1 cell inoculation. Scale bar: 250 μm (upper), 100 μm (lower). (F) IHC image for pSTAT3 of invasive tumor in left sciatic nerve of N‐inv mice. Labeling index of pSTAT3 is calculated in proximal and distal tumor (*n* = 33, *p* = 0.032). (G) IHC image for pSTAT3 in proximal tumor of DMSO group (upper) and AG490 group (lower). More pSTAT3‐positive cells are seen in the DMSO group (*n* = 4) than in the AG490 group (*n* = 6). The pSTAT3 LI is compared between groups (*p* = 0.001). Scale bar: 100 μm. (H) Comparison of xenograft tumor weight between groups (*p* = 0.119)

Figure 2A shows a coronal T_2_‐weighted FSE MRI image of N‐inv model at day 35. Mean longitudinal sizes of the tumor measured on coronal T_2_‐weighted images were 3.20 ± 0.65 mm, 5.55 ± 1.25 mm, and 8.17 ± 1.09 mm at weeks 3, 4, and 5 in three mice, respectively (Figure 2B).

In N‐inv (*n* = 8) and SC mice (*n* = 6), serum levels of IL‐6 and mouse IL‐6 were quantified by ELISA at day 28 after Capan‐1 cell inoculation. Serum level of IL‐6 in N‐inv mice (0.485 ± 0.300 pg/ml) was significantly higher than that in SC mice (0.023 ± 0.038 pg/ml, *p* = 0.004; Figure 2C). Otherwise, no significant differences were identified in serum levels of mouse IL‐6 between N‐inv (0.005 ± 0.009 pg/ml), SC (0.001 ± 0.002 pg/ml), and PBS models (*n* = 4, 0.006 ± 0.012 pg/ml, *F* (2, 15) = 0.524, *p* = 0.603). Normalized IL‐6 mRNA levels in N‐inv tumor (2.289 ± 1.048) were obviously higher than those in SC tumor (0.656 ± 0.512, *p* = 0.002) at day 28 after inoculation (Figure 2D). Obvious expression of IL‐6 protein was observed in proximal sites of N‐inv tumor (Figure 2E). In our N‐inv model (*n* = 33), pSTAT3 expression by tumor nuclei was investigated on day 28. Mean pSTAT3 LI on the proximal side was 7.391 ± 9.734%, higher than mean pSTAT3 LI on the distal side (1.819 ± 2.782%, *p* = 0.032; Figure 2F). To examine the effects of AG490, a JAK2 inhibitor, on pSTAT3 expression and N‐inv, the pSTAT3 LI of tumor nuclei and tumor weight were evaluated on day 28 in N‐inv models treated with AG490 (*n* = 6) and DMSO (*n* = 4). Mean pSTAT3 LI was significantly lower in the AG490 group (1.526 ± 0.924%) than in the DMSO group (9.728 ± 4.184%, *p* = 0.001; Figure 2G). Tumor weight in the AG490 group (0.027 ± 0.020 g) tended to be lower than that in the DMSO group (0.072 ± 0.060 g, *p* = 0.119; Figure 2H). In additional experiment, N‐inv distance in AG490 group (*n* = 7, 2.750 ± 1.614 mm) was significantly shorter than that in the DMSO group (*n* = 7, 5.143 ± 1.380 mm, *p* = 0.011; Figure S7).

### 3.3 Effects of LV‐shRNA‐IL‐6 infection in vitro and in the N‐inv model

**FIGURE 3:**
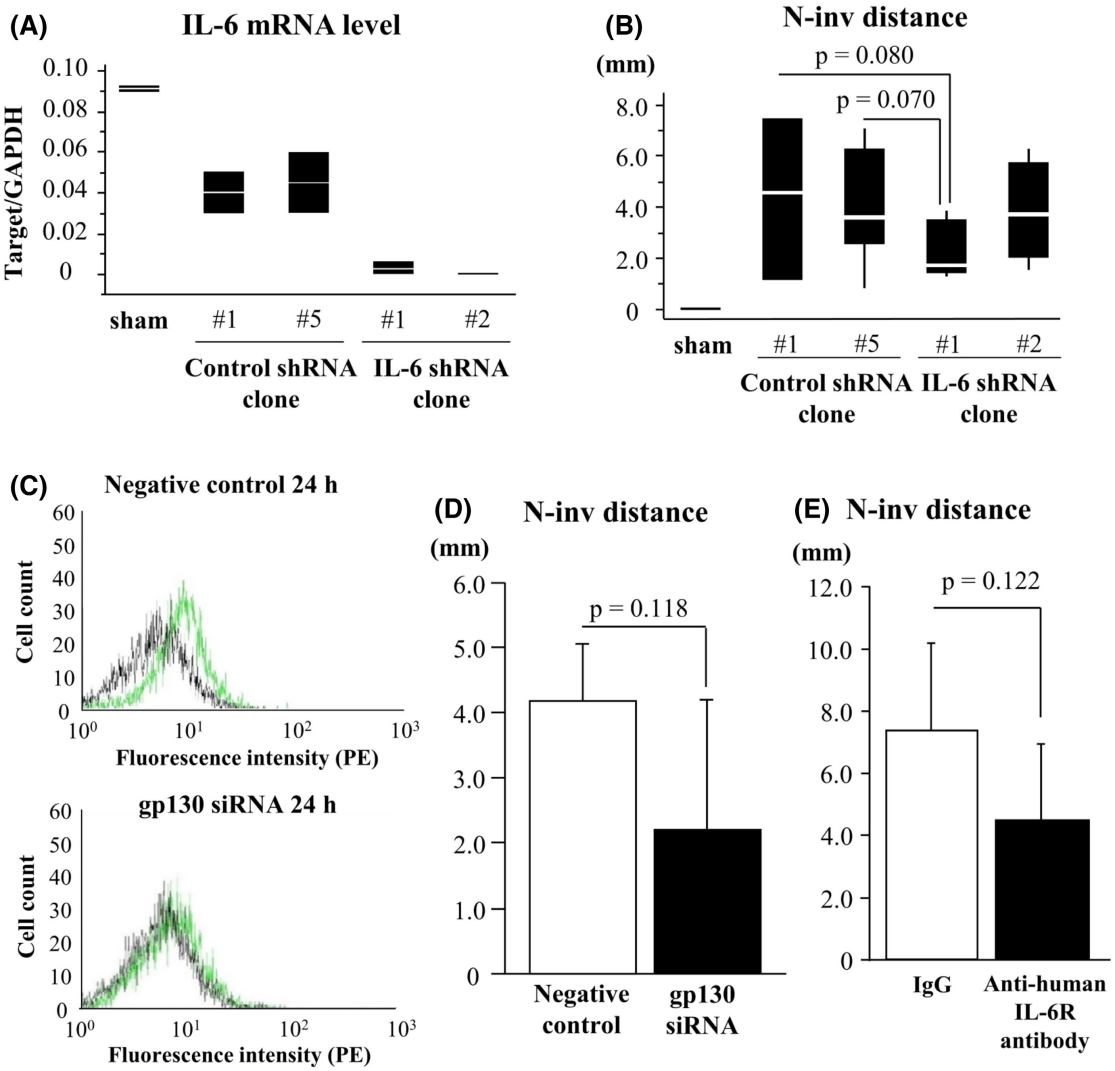
Inhibition of the IL‐6 signaling pathway by RNAi and in a molecule‐specific manner in the N‐inv model. (A) The amount of IL‐6‐knockdown by shRNA transfection is quantified by real‐time RT‐RCR. Expression of IL‐6 mRNA is normalized to that of GAPDH mRNA. (B) N‐inv distance on day 14 after IL‐6 shRNA clone (#1: *n* = 6, #2: *n* = 4) inoculation is compared to that of a control shRNA clone (#1: *n* = 3, #5: *n* = 8, IL‐6 shRNA clone #1 vs. control shRNA clone #5: *p* = 0.070). (C) Cell surface expressions of gp130 protein (in green) analyzed by flow cytometry from Capan‐1‐transfected siRNA negative control (upper) and gp130 siRNA (lower). The percentage of gp130‐positive cells is given for cell population. These results from flow cytometry are shown as representative histograms. (D) N‐inv distance measured 14 days after injecting Capan‐1 transfected siRNA negative control or gp130 siRNA into the left sciatic nerve of mice (each *n* = 5, *p* = 0.118). (E) Comparison of N‐inv distance between the group with antihuman IL‐6R antibody and that with human IgG (*p* = 0.122)

Infection with LV‐shRNA‐IL‐6‐induced marked suppression of IL‐6 mRNA in Capan‐1 compared to LV‐shRNA‐luciferase (mean RNAi rate at 24 h after transfection, 99%) (Figure 3A). Silencing of IL‐6 induced a decrease of N‐inv distance at day 14 after inoculation (IL‐6 shRNA vs. control shRNA [mean]: 1.7 vs. 4.2 mm, *p* = 0.070; Figure 3B).

### 3.4 Cell surface expression and effect of RNAi of IL‐6 receptors in PC cell line

Transfection of siRNAs of IL‐6R or gp130‐induced marked suppression of targeted‐mRNA in Capan‐1 (RNAi rate at 24 h after transfection: IL‐6R, 74%; gp130, 48%; Figure 3C, Figure S3C, D). Silencing of IL‐6R or gp130 of Capan‐1 by RNAi induced a decrease in N‐inv distance (targeted‐RNAi vs. RNAi negative control [mean ± SD]: IL‐6R RNAi 2.719 ± 1.880 mm vs. 4.688 ± 2.459 mm, *p* = 0.250; gp130 RNAi 2.219 ± 1.996 mm vs. 4.206 ± 0.874 mm, *p* = 0.118, Figure 3D).

### 3.5 Effects of anti‐IL‐6R antibody in the N‐inv model

Tocilizumab, anti‐human IL‐6R antibody inhibiting IL‐6‐mediated signaling through both soluble and membrane‐bound IL‐6R,^17^ reduced N‐inv distance at day 21 after inoculation of Capan‐1 cells, compared with IgG (IgG [control] vs. anti‐IL‐6R antibody [mean ± SD], 7.375 ± 2.823 mm vs. 4.479 ± 2.442 mm, *p* = 0.122; Figure 3E). On the other hand, anti‐mouse IL‐6R antibody given by i.p. injection twice a week from day 7 to day 21 after inoculation, did not inhibit tumor growth in the N‐inv model (rat IgG [control] vs. anti‐mouse IL‐6 antibody [mean ± SD], 0.865 ± 0.623 g vs. 1.561 ± 0.755 g; *p* = 0.173). The maximum tumor weight per gram of bodyweight was 9.974%.

### 3.6 N‐inv and serum IL‐6 level in PDAC patients

**FIGURE 4:**
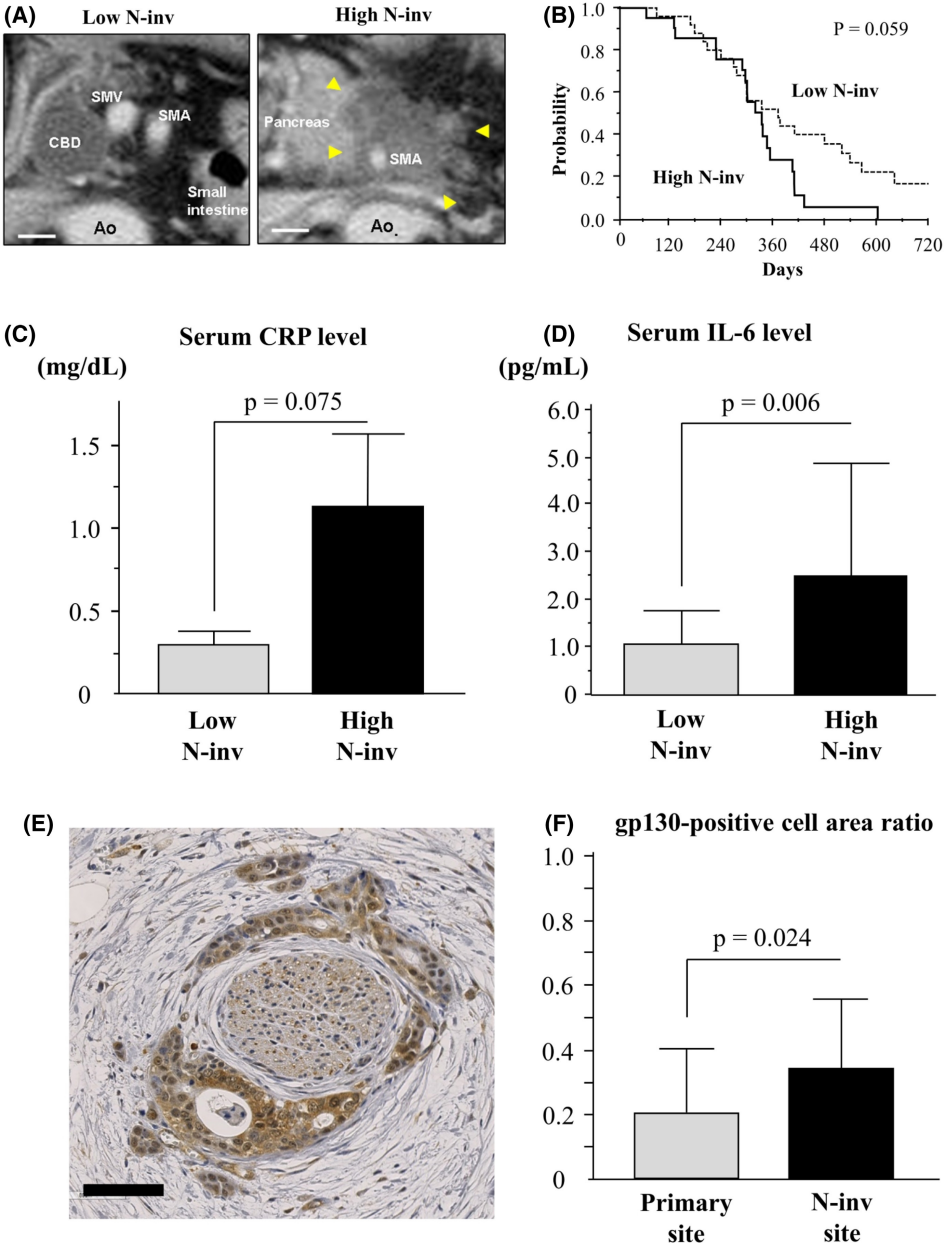
Influence of degree of N‐inv in patients with pancreatic cancer. Patients with PVST that encircled neither the SMA nor CeA completely were assigned to the low N‐inv group. Scale bar: 10 mm. (B–D) Comparison of physical and clinical data before chemotherapy between low N‐inv (*n* = 25) and high N‐inv patients (*n* = 21). Graphs show characteristics of each group such as overall survival (B), serum levels of CRP (C), and serum levels of IL‐6 (D). Results are compared in both groups. (E) IHC images for gp130 of the invasive front at the N‐inv site. Scale bar: 80 μm. (F) Comparison of gp130‐positive cell area ratio in the invasive front between primary sites (*n* = 26) and N‐inv sites (*n* = 24)

The PVST encircling the superior mesenteric or celiac artery on CT images is regarded clinically as radiological N‐inv.^18^ Degrees of N‐inv were assessed using PVST around the SMA and the CeA on CT images from 46 patients with advanced PDAC. Study patients were classified into a high N‐inv group (*n* = 21; 13 women, 8 men; median age, 65 years; range, 55–79 years; primary tumor sites in pancreatic head/body/tail, 10/10/1) and a low N‐inv group (*n* = 25; 15 women, 10 men; median age, 67 years; range, 44–85 years; primary tumor sites in pancreatic head/body/tail, 11/10/4) (Figure 4A). High N‐inv was associated with poor survival (median [95% confidence interval (CI)], high N‐inv 334 days [289–353 days] vs. low N‐inv 372 days [276–519 days]; *p* = 0.059) (Figure 4B) and a high rate of opioid use indicative of severe pain (high N‐inv 33% vs. low N‐inv 16%; *p* = 0.169). In addition, mean serum CRP levels indicative of systemic inflammation tended to be higher in the high N‐inv group (1.136 ± 2.010 mg/ml; *p* = 0.075) than in the low N‐inv group (0.300 ± 0.422 mg/ml) (Figure 4C). Mean serum IL‐6 levels were significantly higher in high N‐inv group (2.512 ± 2.345 pg/ml) than in the low N‐inv group (1.053 ± 0.707 pg/ml; *p* = 0.006, Figure 4D), but no difference in mean IL‐6R levels was evident between groups (high N‐inv, 24.462 ± 6.818 ng/ml vs. low N‐inv, 25.856 ± 7.189 ng/ml; *p* = 0.506). Tumoral gp130 expression was evaluated in resected specimens of human PDAC of pancreas head. The percentage gp130‐positive area was significantly higher in the invasive front of N‐inv sites (*n* = 24, 0.342 ± 0.216) than in that of primary sites (*n* = 26, 0.204 ± 0.199; *p* = 0.024) (Figure 4E, F).

## 4 DISCUSSION

IL‐6 was identified as a stimulator of N‐inv of PC in this study, representing the first evidence of this to our knowledge. Secretion of human IL‐6 produced by Capan‐1 was upregulated in the N‐inv model. When inhibiting synthesis of human IL‐6 from PC cells by RNA silencing and inhibition of human IL‐6/gp130 axis by tocilizumab, anti‐IL‐6R antibody, the growth of tumor was suppressed in the N‐inv model. IL‐6 is a multifunctional cytokine originally identified as a regulator of immune and inflammatory responses.^10^ IL‐6 is associated with cell proliferation, protection of survival, and promotion of cell migration with various cancers, including PC.^16^, ^19^ Human cells only respond to human IL‐6, not to mouse IL‐6.^20^ Several studies have reported on the tumor‐derived IL‐6 in various cancer cells.^21^, ^22^, ^23^, ^24^ This study indicated that the tumor‐derived IL‐6 promoted tumor growth of PC cells.

In our previous studies, high serum levels of IL‐6 were associated with poor survival and high serum levels of CRP in PDAC patients.^17^, ^25^ CRP is an acute‐phase protein that reflects systemic inflammatory conditions.^26^ Transcriptional activation of the CRP gene was upregulated by IL‐6/JAK/STAT3 pathway.^27^, ^28^ High serum IL‐6 levels thus seem to represent high biological activity of IL‐6 in host. In the present research, serum IL‐6 level was elevated in the N‐inv model or PDAC patients with high N‐inv compared with those in the SC model or PDAC patients with low N‐inv (Figures 2D, 4). In addition, tumoral gp130 expressions in N‐inv were significantly higher than those in primary tumor (Figure 4E,F). IL‐6 activates not only the JAK/STAT pathway, but also mitogen‐activated protein kinase pathway via phosphorylation of extracellular‐ regulated kinase (ERK)1 and ERK2.^29^ We have already observed the phosphorylation of ERK1/2 by IL‐6 stimulation in Capan‐1 (Figure S4). ERK2 upregulates the expression of gp130 through binding to the *GP130* promoter, where it conceivably interacts with transcriptional regulatory mechanisms.^30^ Upregulation of gp130 expression indicated high biological activity of IL‐6 signaling, which meant that the IL‐6/gp130 axis in N‐inv was stronger than that in the primary tumor. The N‐inv model mimicked the high biological activity of the IL‐6/gp130 axis in the N‐inv of PDAC.

Inhibition of the IL‐6/JAK/STAT3 pathway suppressed tumor growth in the N‐inv model and could represent a candidate target for the treatment of PDAC. Ruxolitinib and tocilizumab, IL‐6/JAK/STAT3 inhibitors, were tested in PDAC patients. Ruxolitinib is a JAK inhibitor used for the treatment of myelofibrosis and polycythemia vera in clinical practice.^31^, ^32^ Ruxolitinib was investigated in combination with capecitabine in a randomized, double‐blinded, placebo‐controlled phase II study of patients with metastatic PDAC and an inflammatory burden who had experienced failure of gemcitabine therapy.^33^ Ruxolitinib plus capecitabine did not significantly improve overall survival compared with placebo plus capecitabine. Tocilizumab inhibited the dimerization of gp130 and was investigated in combination with gemcitabine in a multicenter, open‐label phase I/II study of patients with PDAC and a high inflammatory burden.^34^ Subpopulation analysis showed that patients with lower baseline CRP levels (<10.6 mg/dl) achieved better survival outcomes than those from archival data of PDAC patients treated with gemcitabine monotherapy. Effective inhibition of IL‐6 signaling was considered to involve not JAK, but rather the IL‐6/gp130 axis in PDAC with an inflammatory burden.

The perineural space into which tumor cells invade is filled with cerebrospinal fluid containing various neurotrophic factors.^35^, ^36^, ^37^, ^38^ Cytokines including IL‐6 are transported from blood vessel into the subarachnoid space via epithelial cells and stromal cells in the choroid plexus.^39^ At the invasive front of the tumor, intraneural tumor clogged the perineural space, and dammed cerebrospinal fluid. IL‐6 contained in cerebrospinal fluid could be enriched at the invasive front. Moreover, tumor cells in the perineural space usually attach to endoneurium and perineurium and are surrounded by Schwann cells and fibroblasts.^40^, ^41^ IL‐6 is known to be secreted from tumor cells, cancer‐associated fibroblasts,^42^ and Schwann cells.^43^ In our model, obvious expression of mouse IL‐6 protein was observed in Schwann cells at proximal sites of N‐inv tumor (Figure S5A–C). In the clinic, tumor cells of N‐inv could be promoted by IL‐6 from both tumor cells and Schwann cells.

This study had several limitations. The relationship between cytoplasmic gp130 expression and biological activity of IL‐6 signaling could involve an increase in soluble gp130 which would neutralize IL‐6/sIL‐6R complexes.^10^ Soluble gp130 was constitutively present in the upper nanogram/milliliter range,^44^ but few reports have evaluated soluble gp130 in cancers. Further study is needed to investigate relationships between activity of the IL‐6/gp130 axis and serum levels of soluble gp130. Recently, LIF, a member of the IL‐6 superfamily, was found to promote neural remodeling^45^ and tumor growth in PC.^46^ Stimulators of N‐inv were speculated to include not only IL‐6, but also LIF. This study did not focus on LIF, since the stimulant activity of LIF on PC cells is not dependent on the dosage of LIF. Our N‐inv model used Capan‐1 cells as cancer cells, but MiaPaCa‐2 cell, human PC cell, showed modest neural invasion in our previous report^11^ and upregulated LIF in tumor microenvironment in a paracrine manner.^45^ When investigating N‐inv models using MiaPaCa‐2, LIF would be annotated as a stimulator of N‐inv. Generally, resectability in the NCCN guidelines^15^ is used to evaluate the degree of neural invasion around the superior mesenteric or celiac artery on CT images in patients with PC. However, we have not evaluated the association between serum IL‐6 or CRP level and resectability based on the NCCN guidelines, since PVST encircling the superior mesenteric or celiac artery on CT images sufficiently reflected clinical radiological N‐inv.^4^, ^18^

In conclusion, the present study revealed IL‐6 as a stimulator of N‐inv in PC. Thus, the IL‐6/gp130/JAK/STAT3 pathway could be a promising target for therapeutic intervention for PDAC.

## CONFLICT OF INTEREST

The authors declare that they have no conflict of interest.

## AUTHOR CONTRIBUTIONS

H. Suzuki substantially contributed to conception and design, data acquisition, analysis and interpretation, and drafting of this manuscript. Mitsunaga substantially contributed to conception and design, data acquisition, analysis and interpretation, and critical revisions of this manuscript for important intellectual content. Ikeda, Aoyama, Yoshizawa, Yamaguchi, M. Suzuki, Narita, Kawasaki, and Ochiai substantially contributed to the conception and design of the study, and the analysis and interpretation of the data. Ochiai also revised this manuscript critically for important intellectual content. All authors approved the submitted manuscript for publication.

## ETHICS APPROVAL AND CONSENT TO PARTICIPATE

All human studies were approved by the National Cancer Center Ethics Committees, and only patients from whom written informed consent had been obtained were examined (K2011‐001). All animal experiments were approved by Committee for Ethics of Animal Experimentation of the National Cancer Center (K17‐001).

## Supporting information


Table S1

Table S2

Figure S1

Figure S2

Figure S3

Figure S4

Figure S5

Figure S6

Figure S7

Appendix S1

## Data Availability

The datasets used and/or analyzed during the present study are available from the corresponding author upon reasonable request.
