# Supplementary figures and images for: Interleukin 6/gp130 axis promotes neural invasion in pancreatic cancer
Source: Cancer Med. 2022 May 16;11(24):5001–12. doi: 10.1002/cam4.4823 (PMC9761092; doi:10.1002/cam4.4823)

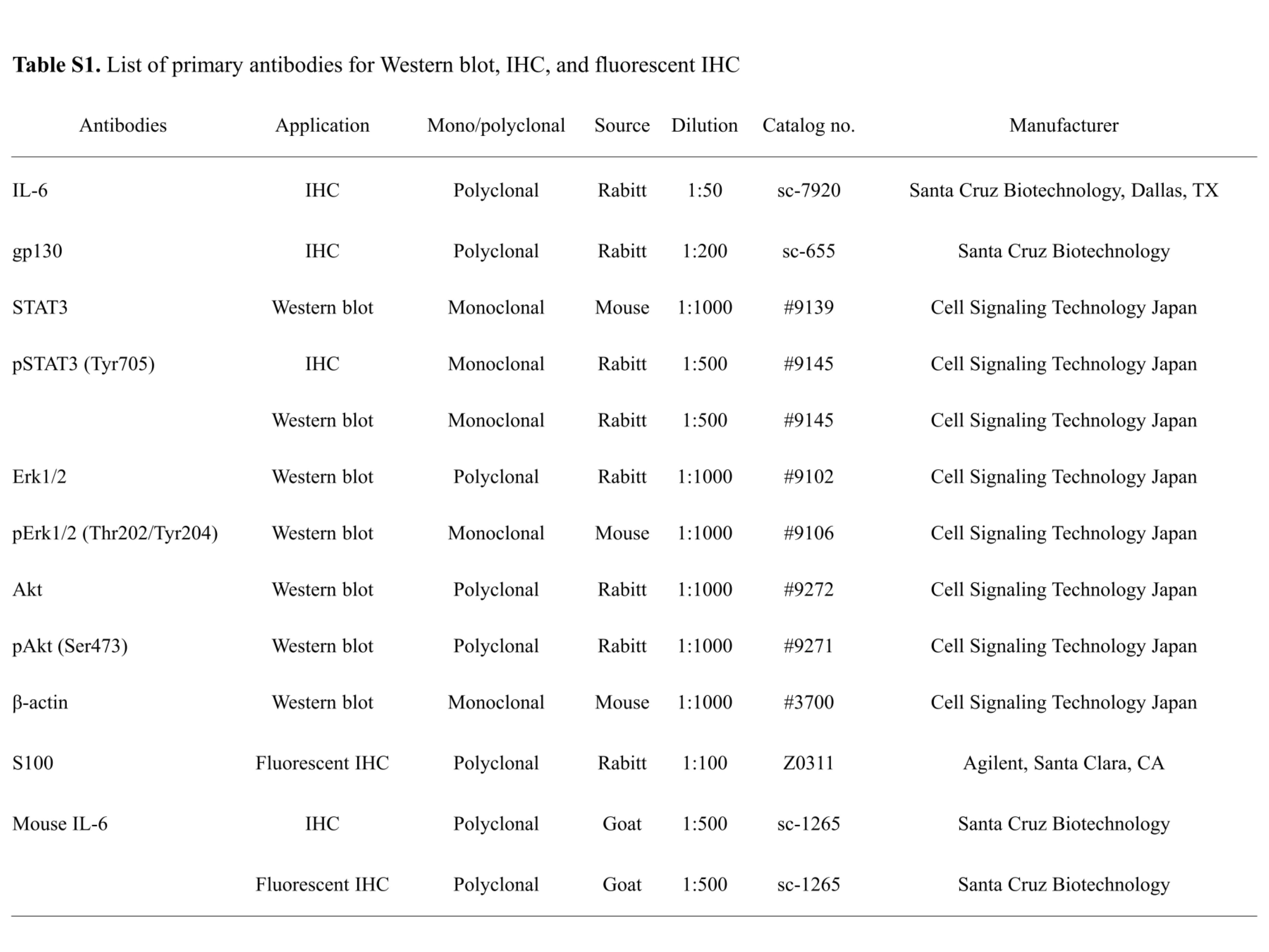

Supplement: Supplementary file 1 — Table S1 [file CAM4-11-5001-s004.jpg]

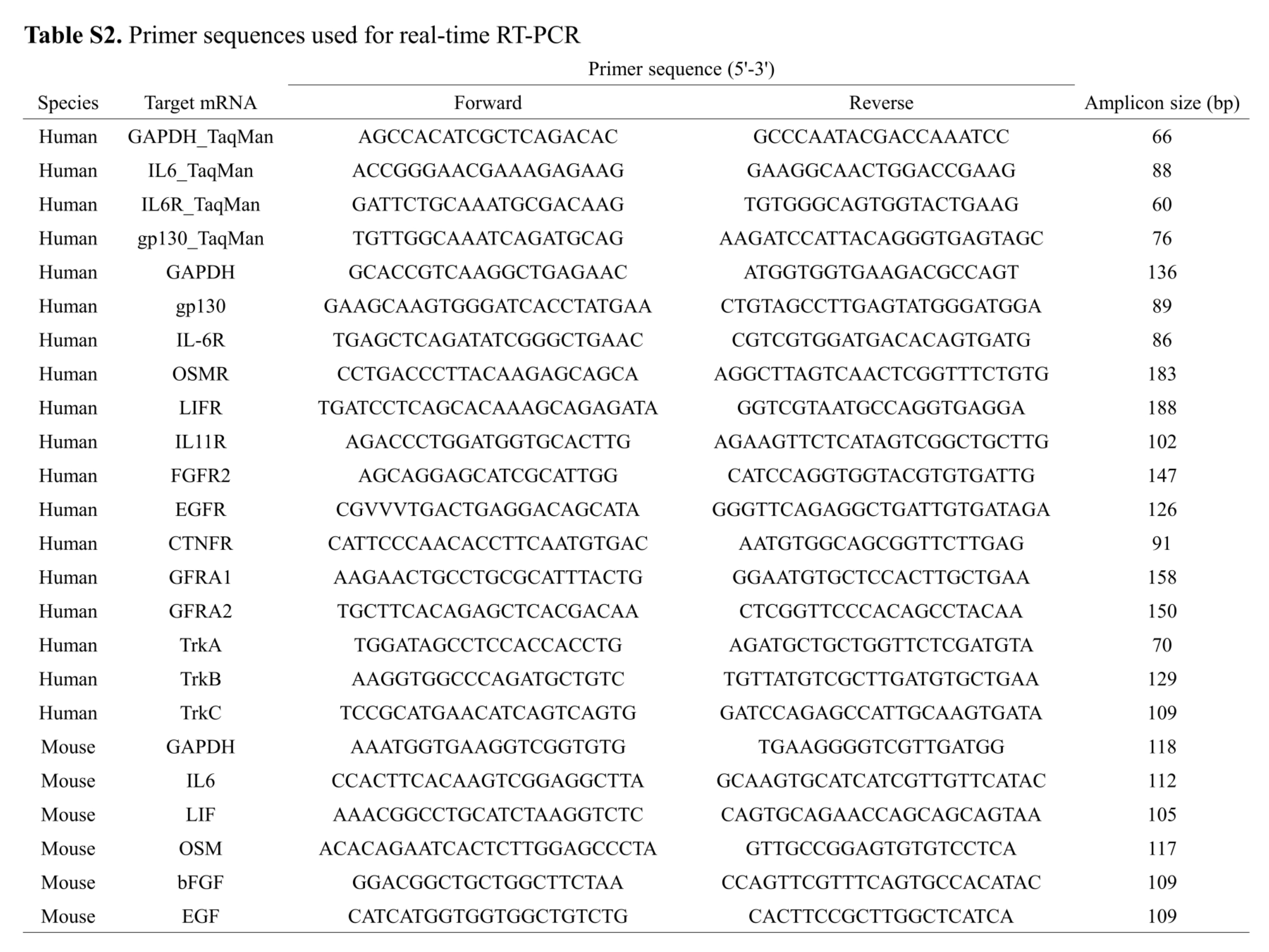

Supplement: Supplementary file 2 — Table S2 [file CAM4-11-5001-s001.jpg]

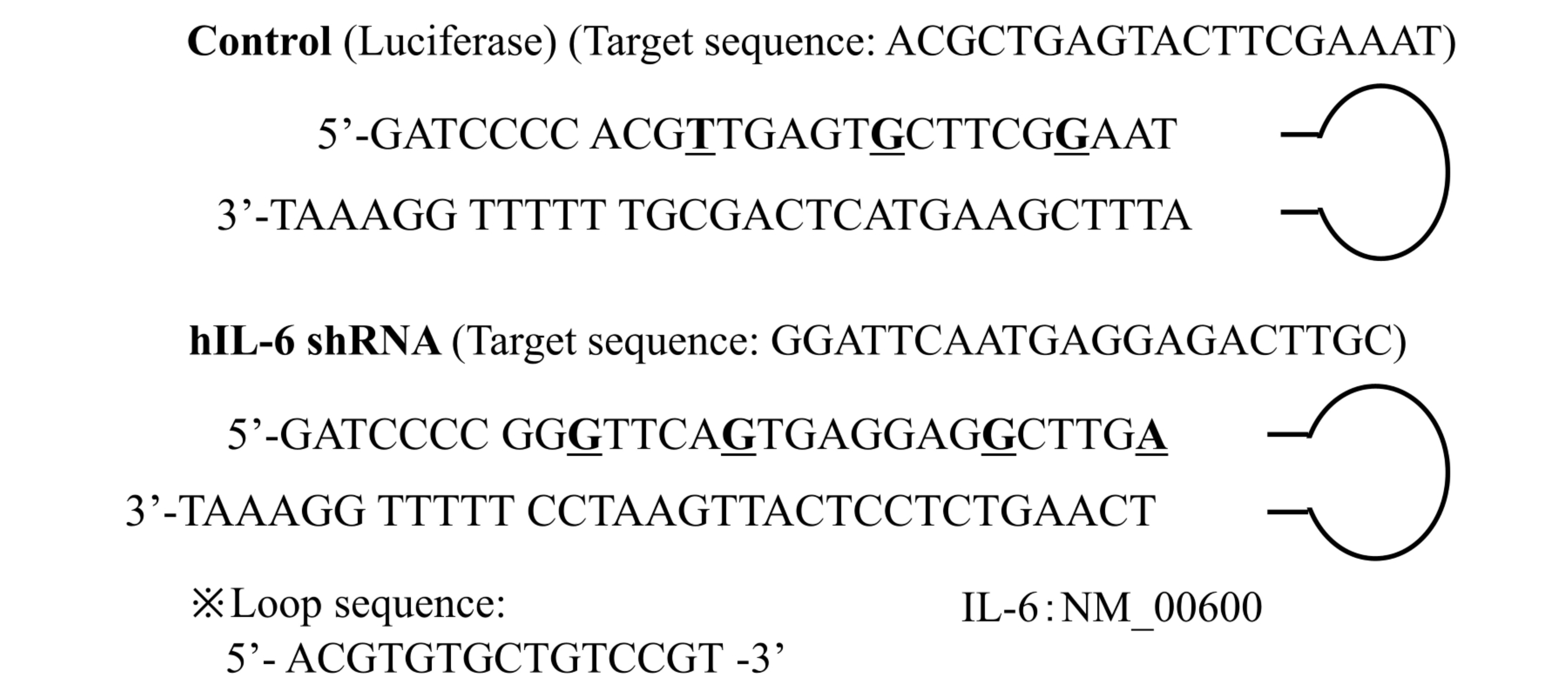

Supplement: Supplementary file 3 — Figure S1 [file CAM4-11-5001-s005.jpg]

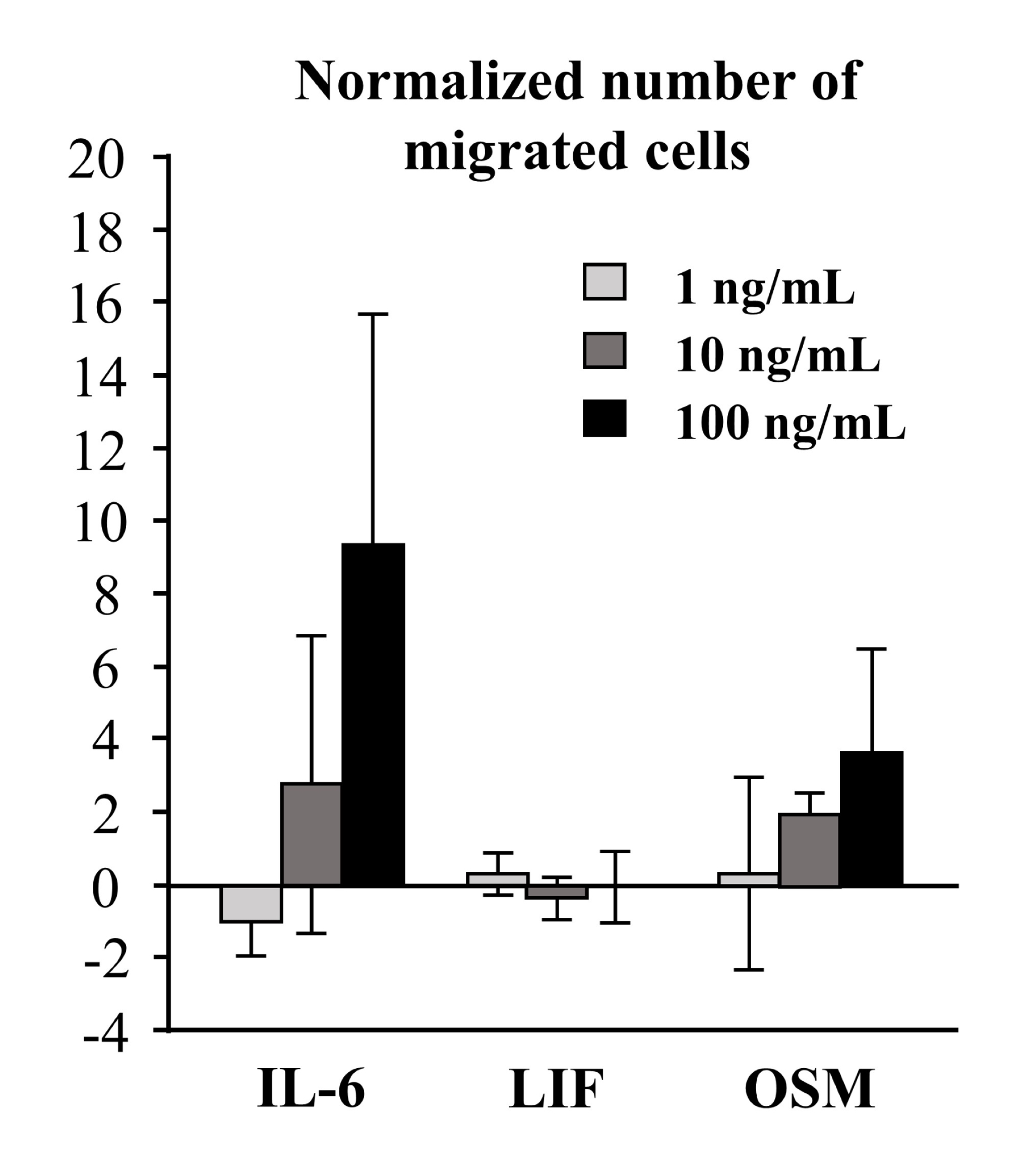

Supplement: Supplementary file 4 — Figure S2 [file CAM4-11-5001-s002.jpg]

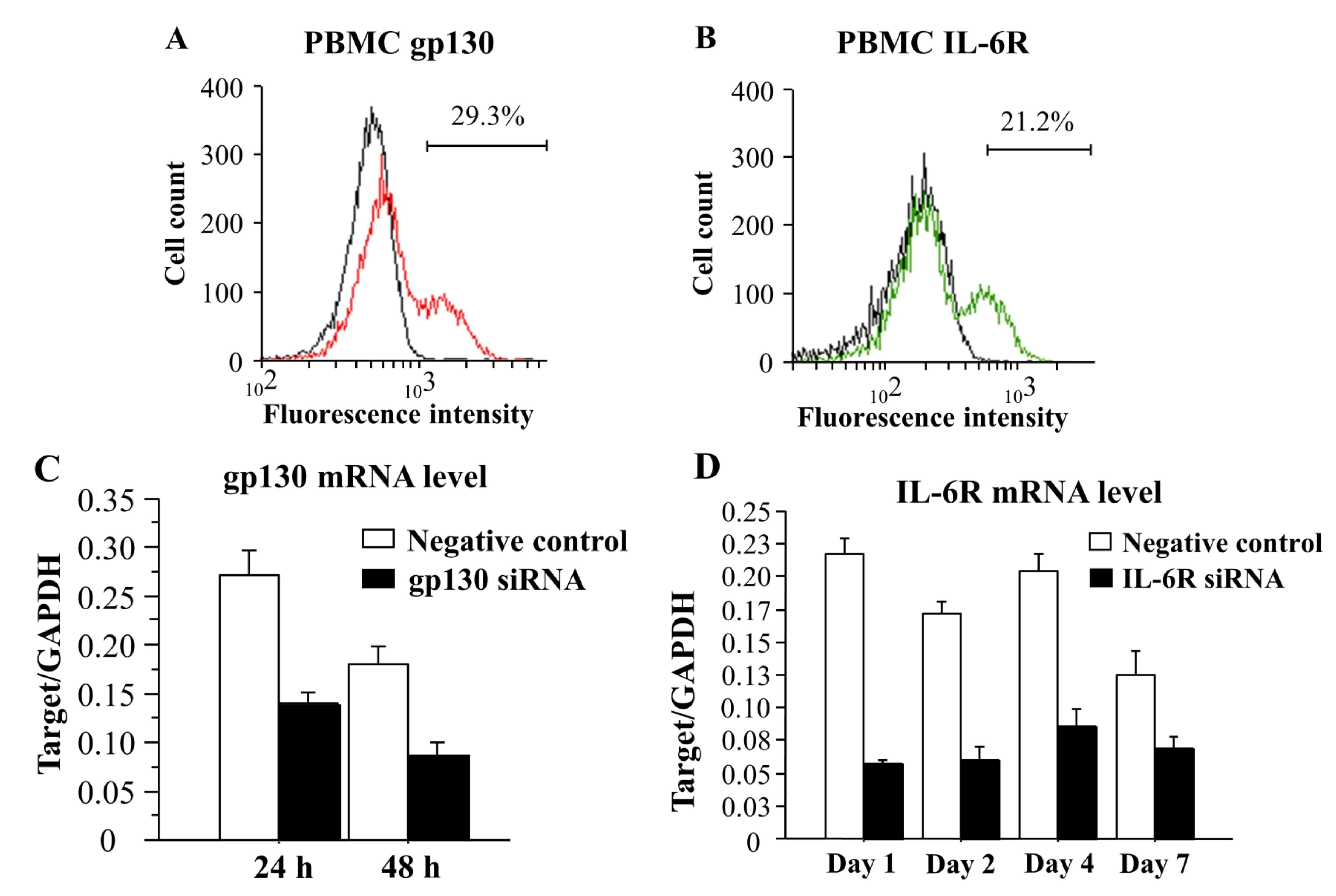

Supplement: Supplementary file 5 — Figure S3 [file CAM4-11-5001-s010.jpg]

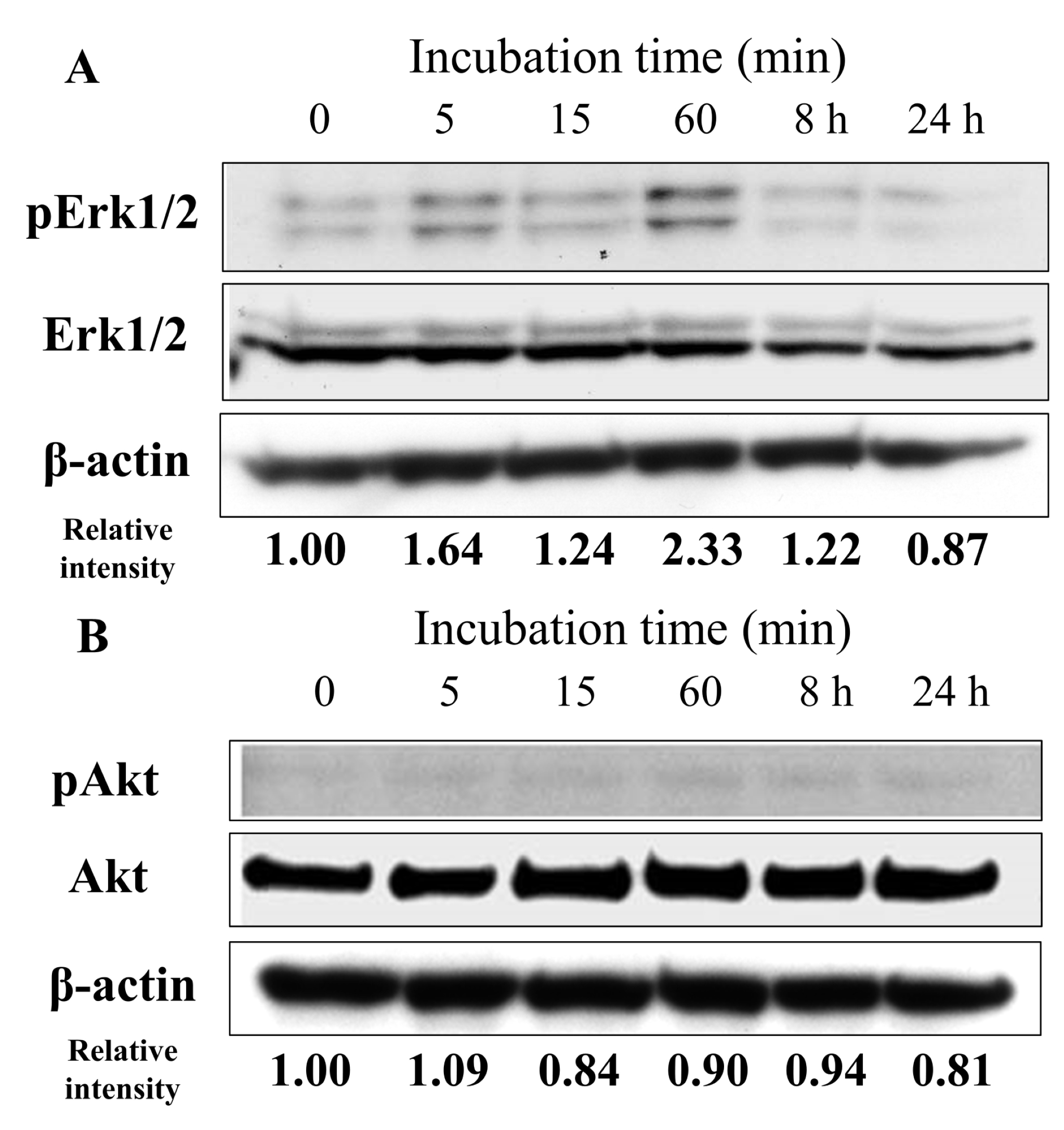

Supplement: Supplementary file 6 — Figure S4 [file CAM4-11-5001-s006.jpg]

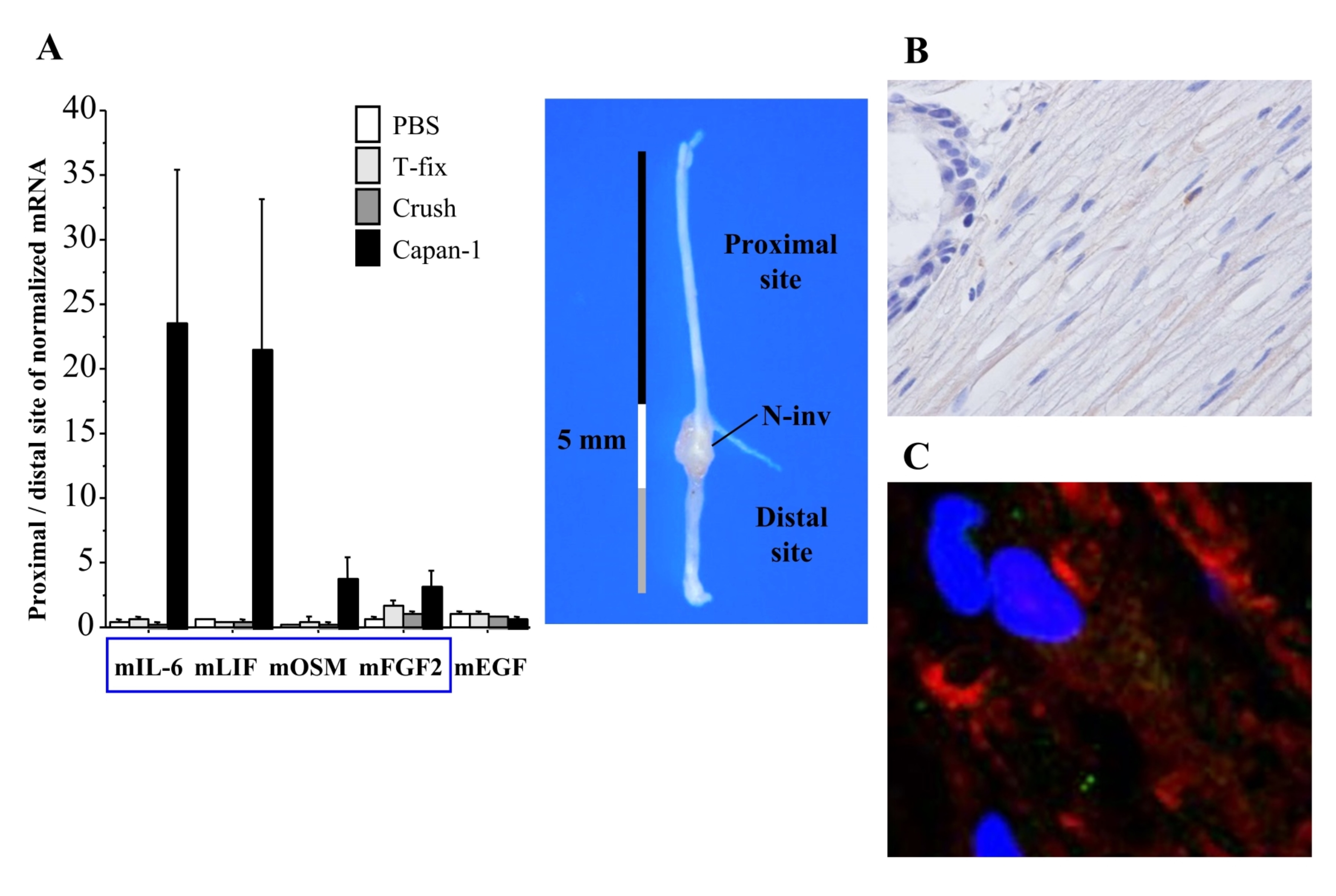

Supplement: Supplementary file 7 — Figure S5 [file CAM4-11-5001-s007.jpg]

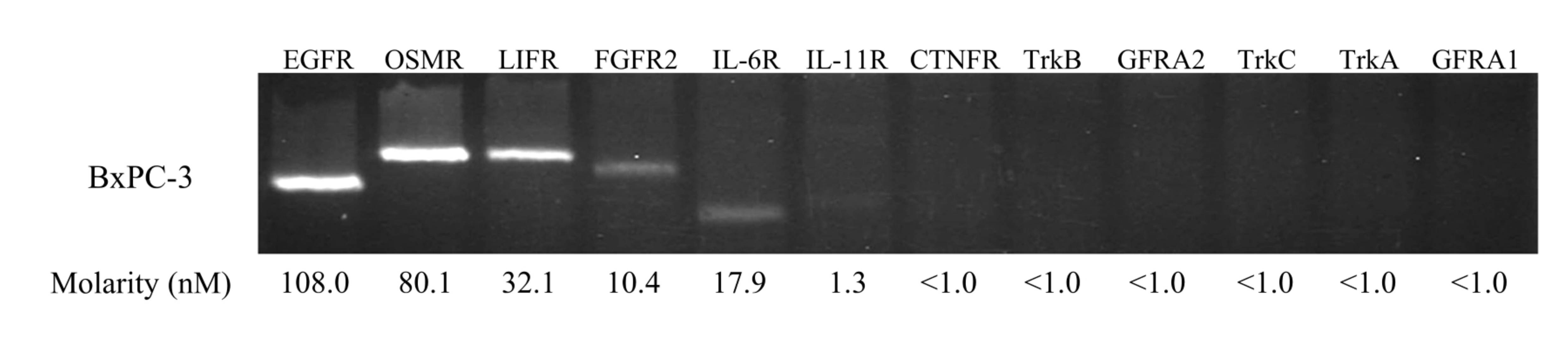

Supplement: Supplementary file 8 — Figure S6 [file CAM4-11-5001-s008.jpg]

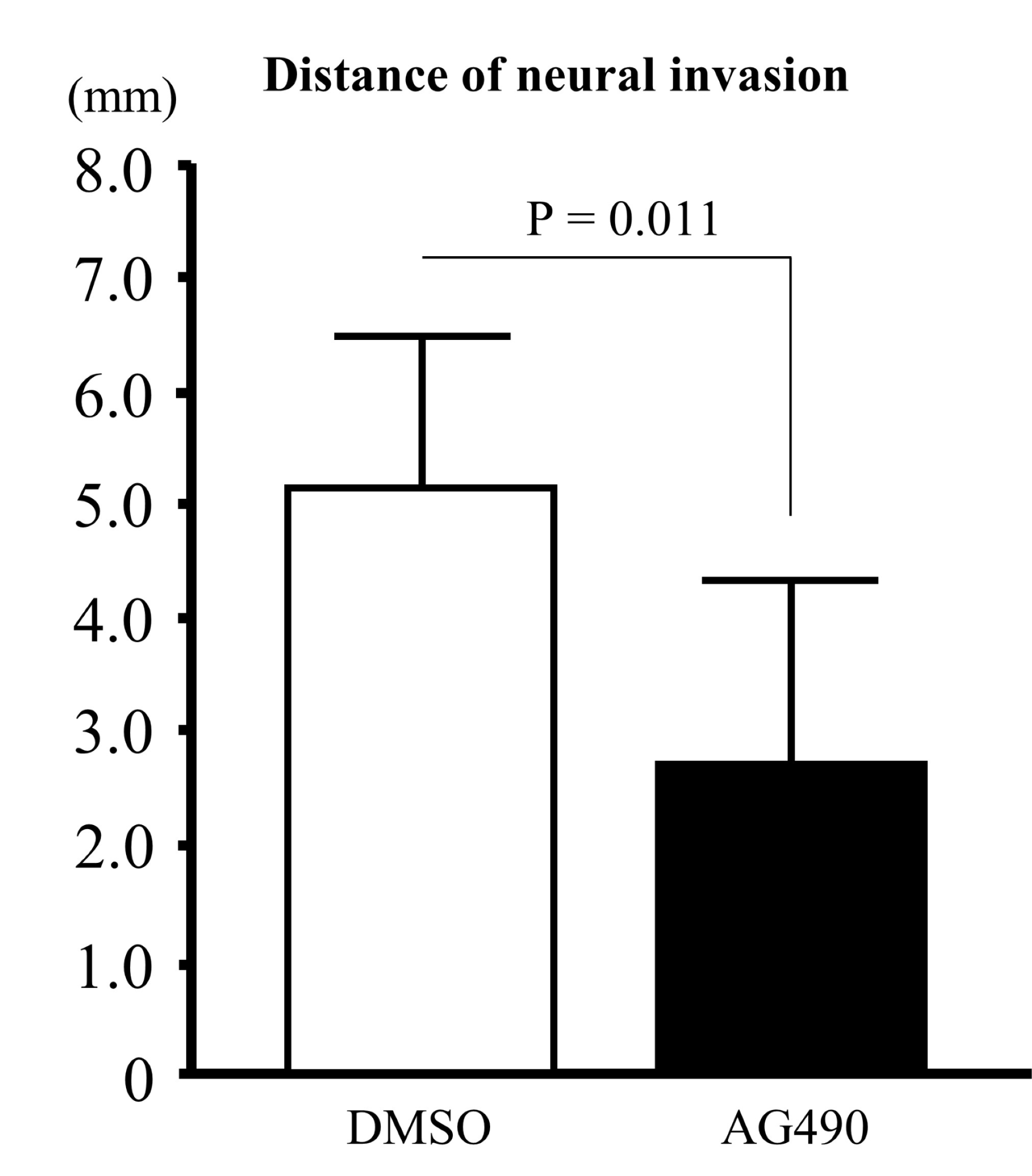

Supplement: Supplementary file 9 — Figure S7 [file CAM4-11-5001-s003.jpg]
