# Supplementary material for: Interleukin 6/gp130 axis promotes neural invasion in pancreatic cancer
Source: Cancer Med. 2022 May 16;11(24):5001–12. doi: 10.1002/cam4.4823 (PMC9761092; doi:10.1002/cam4.4823)
Supplement: Supplementary file 10 — Appendix S1 [file CAM4-11-5001-s009.docx]

# **Doc. S1**

# **Supplementary methods**

## **IHC**

After microwave heating in 0.01-M Tris-ethylenediaminetetraacetic acid (EDTA) buffer (pH 7.5), slides were incubated with anti-mouse IL-6 antibody (Table. S1) at room temperature for 30 min.

## **Fluorescent IHC**

After microwave heating in 0.01-M Tris-EDTA buffer (pH 7.5), slides were incubated with a mixture of anti-mouse IL-6 antibody and anti-S100 antibody (Table. S1) at room temperature for 1 h. These slides were washed out in PBS and incubated with a mixture of chicken anti-goat IgG (Alexa Fluor^®^ 488; Thermo Fisher Scientific) and donkey anti-rabbit IgG (Alexa Fluor^®^ 555; Thermo Fisher Scientific) at room temperature for 30 min. Nuclei were stained with DRAQ5 (1:1000; Cell Signaling Technology Japan). Photographs were taken under confocal microscopy (LSM 5 PASCAL; Carl Zeiss, Tokyo, Japan).

## **Mouse model experiment**

The N-inv model using Capan-1 was produced as *Methods* in manuscript. Briefly, 6-week-old SCID mice were used in our study. After induction of anesthesia with 4-5% isoflurane in oxygen (O_2_), mice were maintained in 2-3% isoflurane anesthesia via a nose cone throughout the operation. After the left and right sciatic nerves of each mouse was exposed at the level of femur, 2.5 μL of phosphate buffered saline (PBS) containing 2.5 ×10^4^ Capan-1 cells was injected into the both sciatic nerves using a micro-syringe (Hamilton, Reno, NV) and 30-gauge needle (Becton Dickinson and Company, Franklin Lakes, NJ). AG490, an inhibitor of JAK2, at a dose of 0.5 mg/kg (Calbiochem, San Diego, CA; n = 4) or dimethyl sulfoxide (DMSO) vehicle (1%/1 mL/body, n = 4) was administered to N-inv mice by intraperitoneal (i.p.) injection from day 3 to day 14, daily. After euthanasia with continued exposure to 5% of isoflurane until respiration ceases and death ensues, mice were sacrificed 2 h after the last dose.
